# Using Stable Isotopes of Carbon and Nitrogen to Mark Wild Populations of *Anopheles* and *Aedes* Mosquitoes in South-Eastern Tanzania

**DOI:** 10.1371/journal.pone.0159067

**Published:** 2016-07-08

**Authors:** Mercy A. Opiyo, Gabriel L. Hamer, Dickson W. Lwetoijera, Lisa D. Auckland, Silas Majambere, Fredros O. Okumu

**Affiliations:** 1 Environmental Health and Ecological Sciences Thematic Group, Ifakara Health Institute, Ifakara, Tanzania; 2 Vector Biology Department, Liverpool School of Tropical Medicine, Liverpool, United Kingdom; 3 Department of Entomology, Texas A&M University, College Station, Texas, United States of America; 4 Department of Veterinary Integrative Biosciences, Texas A&M University, College Station, Texas, United States of America; 5 Innovative Vector Control Consortium, Liverpool, United Kingdom; 6 School of Public Health, Faculty of Health Sciences, University of the Witwatersrand, Parktown, Republic of South Africa; Instituto de Higiene e Medicina Tropical, PORTUGAL

## Abstract

**Background:**

Marking wild mosquitoes is important for understanding their ecology, behaviours and role in disease transmission. Traditional insect marking techniques include using fluorescent dyes, protein labels, radioactive labels and tags, but such techniques have various limitations; notably low marker retention and inability to mark wild mosquitoes at source. Stable isotopes are gaining wide spread use for non-invasive marking of arthropods, permitting greater understanding of mosquito dispersal and responses to interventions. We describe here a simple technique for marking naturally-breeding malaria and dengue vectors using stable isotopes of nitrogen (^15^N) and carbon (^13^C), and describe potential field applications.

**Methods:**

We created man-made aquatic mosquito habitats and added either ^15^N-labelled potassium nitrate or ^13^C-labelled glucose, leaving non-adulterated habitats as controls. We then allowed wild mosquitoes to lay eggs in these habitats and monitored their development *in situ*. Pupae were collected promptly as they appeared and kept in netting cages. Emergent adults (in pools of ~4 mosquitoes/pool) and individually stored pupae were desiccated and analysed using Isotope Ratio Mass Spectrometry (IRMS).

**Findings:**

*Anopheles gambiae* s.l and *Aedes* spp. from enriched ^13^C and enriched ^15^N larval habitats had significantly higher isotopic levels than controls (P = 0.005), and both isotopes produced sufficient distinction between marked and unmarked mosquitoes. Mean δ^15^N for enriched females and males were 275.6±65.1 and 248.0±54.6, while mean δ^15^N in controls were 2.1±0.1 and 3.9±1.7 respectively. Similarly, mean δ^13^C for enriched females and males were 36.08±5.28 and 38.5±6.86, compared to -4.3±0.2 and -7.9±3.6 in controls respectively. Mean δ^15^N and δ^13^C was significantly higher in any pool containing at least one enriched mosquito compared to pools with all unenriched mosquitoes, P<0.001. In all cases, there were variations in standardized isotopic ratios between mosquito species.

**Conclusion:**

Enrichment of semi-natural mosquito larval habitats with stable isotopes of nitrogen and carbon resulted in effective marking of *Anopheles* and *Aedes* mosquitoes colonizing these habitats. This approach can significantly enhance studies on mosquito eco-physiology, dispersal, pathogen transmission and responses to control measures.

## Background

Marking insect vectors in the field is crucial for understanding their various ecological components such as dispersal range, population density, food sources and population dynamics. For example, marking and following movement of mosquitoes can provide useful reference information for epidemiological surveys for mosquito-borne disease cases. In addition, accurate information on mosquito bionomic parameters is not only crucial when designing vector control strategies, but also for successful implementation of control measures against malaria vectors [1,2]. Biologists have utilized different techniques, including mark, release and recapture (MRR) to estimate flight range, feeding patterns, longevity and gonotrophic cycles of insect vectors [2]. Extrinsic markers such as dyes, paints, dusts [3] and intrinsic labels such as trace elements [4–6] and radio-active elements [7] have been intensively used to mark insects, with varying successes. The ultimate marking techniques should not be harmful to users, the environment or the target insects, should be used with ease, should be cost effective, should last the entire life span of the insect, should be applicable both in the field and laboratory studies, and should require simple methods of analysis [2].

Most existing techniques for marking mosquitoes are time consuming, requiring large numbers of mosquitoes to be reared, marked, released and recaptured. Consequently, the handling process may render them different compared to the wild population [3]. For example, marking mosquitoes using fluorescent dusts, dyes and even paints require that they are first immobilised either by freezing, a process which has significant impact on their survival as well as fitness [3]. A previous study has also shown that using fluorescent dyes or powders for marking mosquitoes that were at least 5 days old reduced their daily survival probabilities [8]. Most importantly, the commonly-used techniques cannot mark large mosquito populations at source without constructing special structures and majority often require captive specimen. For example, Ciota *et al* demonstrated marking egressing *Culex* spp. using fluorescent dust in the USA using a self-marking device [9].

Recently, naturally occurring stable isotopes have been utilized for use in mosquito studies because these isotopes can mark wild mosquitoes at source and can persist across the different mosquito life cycle stages [4], are environmentally safe and easy to handle, and do not require sophisticated techniques other than the isotopic analysis process itself [10,11]. Stable isotopes have also been intensively utilized in ecological [12,13]and agricultural studies [14] as useful tracers for organisms, their foods, the parasites they transmit, their mating behaviours and their positions in food webs and trophic levels [10]. For example, stable isotopes have been used to investigate division of food resources among co-existing larval species in mosquito aquatic habitats [15] and to estimate the proportion of insect host plants [16].

Isotopes of an element have the same number of protons but different numbers of neutrons. In other words, they are forms of the same element, having same atomic numbers, but different mass numbers. Stable isotopes occur naturally in the environment, are non-toxic, non-radioactive and incorporate very well into the living tissues of organisms [10,11]. Examples of elements with naturally occurring stable isotopes include carbon, nitrogen, oxygen and hydrogen. In the environment, atoms of stable isotopes of ^15^N and ^13^C are approximately 0.4% and 1%, the rest being 99.6% and 99% ^14^N and ^12^C respectively.

As reviewed by Hood-Nowotny and Knols [10], using stable isotopes in ecological studies may involve: 1) enrichment studies (addition of enriched compounds containing desired stable isotopes, e.g. introducing fertilizers that are highly enriched with ^15^N into insect breeding habitats to mark the insects or garden plots to mark plants), 2) natural abundance studies (identifying and monitoring natural differences in ratios of isotopic signatures in organism or environments, and using these differences to track sources and end points of organic material), or 3) a combination of the two approaches. Isotope enriched compounds are commercially available and their costs are reducing. Moreover, technologies for isotopic analyses such as mass spectrometry and laser are becoming increasingly scalable and unit costs of analysis are now comparable to most chemical and molecular analyses used by entomologists, such as polymerase chain reaction (PCR) [11].

Enriched compounds of ^13^C and ^15^N have been previously evaluated to mark mosquitoes i.e. *Culex* in the laboratory and in the field with great success [4,13]. Carbon isotopes, ^13^C have also been tested for marking the malaria vector, *Anopheles arabiensis* in laboratory settings, demonstrating persistence for up to 21 days after emergence [12] and to study mating success in the same vector species [17]. Hamer *et al* utilized stable isotopes for field marking of wild mosquitoes at source and demonstrated long-lasting marking (detectable up to 55 days post-emergence) of wild *Culex pipiens* mosquitoes in USA [4], later using the same technique to study flight range of the same vector in a geographical foci of West Nile virus transmission [13]. To our knowledge, stable isotope marking of malaria vectors in natural habitats has not been pursued.

The aim of this study was to demonstrate use of stable isotope enrichment technique for marking naturally breeding malaria and dengue vectors at source, in rural Tanzania. This study builds on, and uses similar methodologies of previous studies on *Culex pipiens* mosquitoes in USA [4,13]. We used enriched commercially available ^15^N-labelled potassium nitrate (KNO_3_; ^15^N, 99 atom%) and ^13^C-labelled glucose (U-^13^C_6_, 99 atom%), incorporated into feeding regimes of mosquitoes in man-made larval habitats in which wild mosquitoes laid their eggs. The study was therefore also aimed at providing the first set of field-evidence for marking naturally breeding Afro-tropical malaria vectors, *Anopheles gambiae* complex.

## Materials and methods

### Study site and basic experimental set-up

The study was conducted at Kining’ina village within a plot of land where the Ifakara Health Institute Semi-Field Experimental Facilities (*The Mosquito City*) are also located (8.11417 S, 36.67484 E). This village is 5 km north of Ifakara town, in south-eastern Tanzania. Two rounds of experimentation, each with six replicates, were completed. The first round of experiments were ran between 15^th^ August and 11^th^ September, 2015, while the second round was run between 28^th^ September and 31^st^ October, 2015, using a protocol adapted, with modifications, from previous studies using isotopes for insect marking [4,15,18]. Each experiment was conducted for 4 weeks.

**Fig 1:**
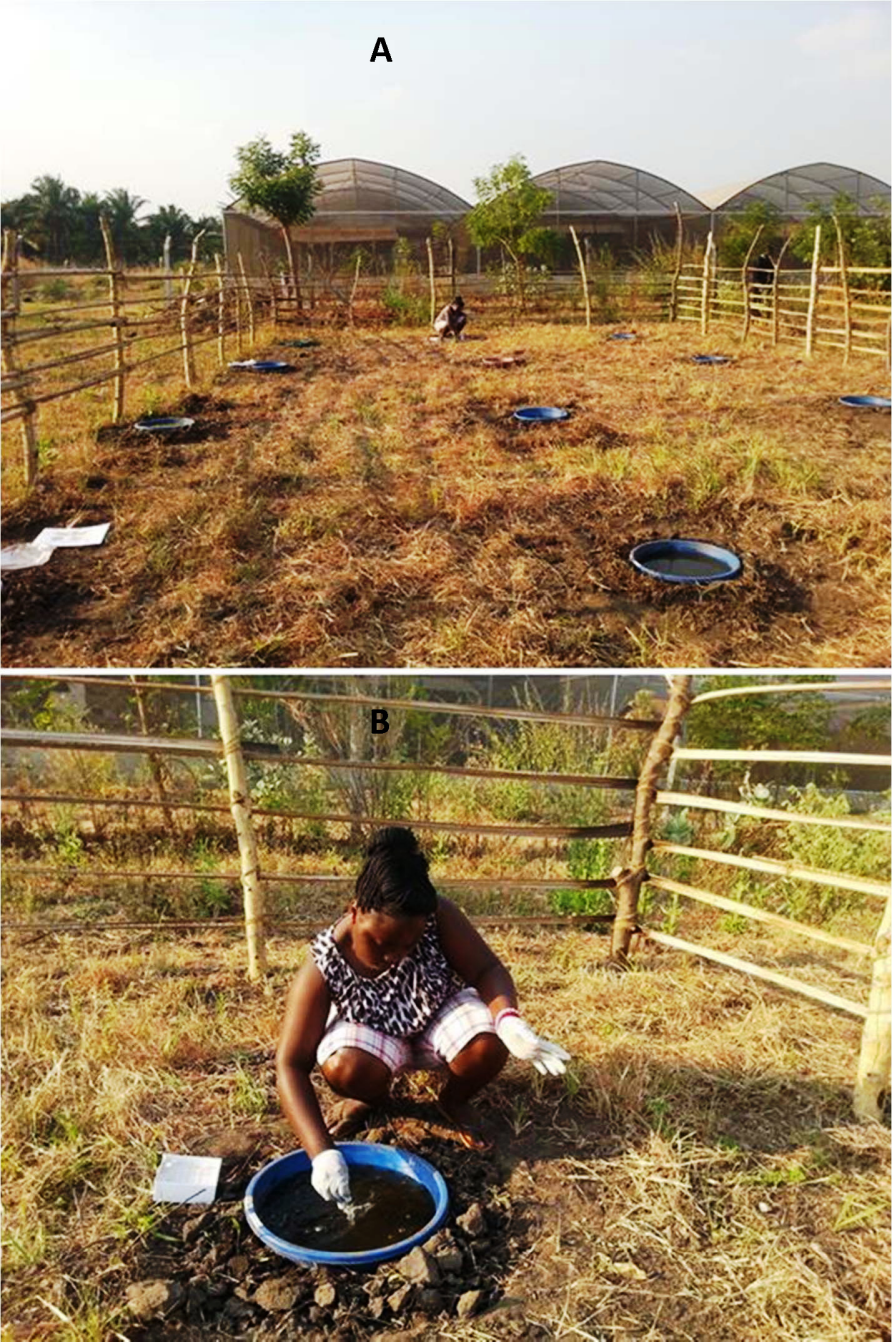
**Experimental set up:** Showing artificial mosquito breeding habitats created using wash basins (A), and the process of treating breeding habitats with stable isotopes (B).

Using locally purchased round plastic wash basins (50cm diameter with a holding capacity of 15 litres each), we established an array of 18 artificial mosquito aquatic habitats to mimic local natural larval habitats in the region. The basins were set 3m apart in an open sunlit area covering a 36m^2^ areas (Fig 1). Approximately 2 kg of local top-soil was added into each basin to increase microorganism colonisation as well as to maintain cooler temperatures in the habitats. The soil used was dug from the same locations as the basins, such that the basins were actually sunk into the resulting depressions to ground level (Fig 1). We thoroughly mixed the soil to achieve uniformity across all the breeding habitats. Eight litres of rain water collected by trapping run-off water from the nearby screen house roofing gutters, was added into each basin. After adding the water, all wash basins were covered with netting mesh, to avoid oviposition by wild mosquitoes, and left for 8 days for microorganism colonization, prior to enrichment with stable isotopes.

### Enrichment with stable isotopes

The stable isotopes used for enriching the breeding habitats in this study were ^15^N-labelled potassium nitrate (KNO_3_; ^15^N, 99 atom%; Cambridge Isotope Laboratories, Inc., Andover, MA) and ^13^C-labelled glucose (U-^13^C_6_, 99 atom%; Cambridge Isotope Laboratories, Inc., Andover, MA). We randomly selected six wash basins to receive ^15^N-labelled potassium nitrate, six others to receive ^13^C-labelled glucose and the remaining six as controls, in which no enrichment was done. The untreated wash basins were used to determine natural abundance of stable isotopes. Unlike in the original laboratory studies by [4], we did not determine the amount of ^15^N and ^13^C in the wash basins. Instead, we calculated the amount of stable isotopes on the basis of the volume of water we added into each wash basin. We aimed to achieve a target dose of 0.002g of ^15^N or ^13^C per litre of water, using 0.01g isotope/ml solutions made using deionized water as this target dose has been shown to be sufficient to differentiate marked mosquitoes from unmarked ones without any harmful effect on mosquitoes, and with no transgenerational marking with stable isotope [4]. Therefore for the 8 litres of water in each wash basin, we used a total of 0.016g in a solution of 1.6ml of ^15^N or ^13^C for the initial enrichment for each wash basin. This initial enrichment was done on 21^st^ August and 4^th^, October, 2015 for first and second rounds of experiments respectively, after which the basins were left open to allow wild mosquitoes to lay eggs.

During this experimental period, there was no rainfall recorded and hence dilution and flushing did not occur in the prepared wash basins. The ground area around each of the wash basins was also watered for the period of the experiment in an attempt to maintain cooler temperatures and mimic the natural aquatic habitat surroundings. For the entire 4 weeks of each experiment, we added re-enriched water twice in the first experiment (on the 4^th^ September, 2015 and on the 9^th^ September, 2015), and in the second experiment once (on the 15^th^, October, 2015). The total amount of stable isotopes used to enrich the basins for the entire experiment one was 0.156 g of ^15^N-labelled potassium nitrate and 0.156 g of ^13^C-labelled glucose. For the second round, we used a total of 0.144 g of ^15^N-labelled potassium nitrate and 0.144g of ^13^C-labelled glucose. We then monitored larval development in each wash basin on a daily basis from the initial day of enrichment. In the first round of tests, early instar larvae were first seen on day 6 in one basin enriched with potassium nitrate. Pupae first appeared on day 13 in nearly three-quarters of the wash basins. In the second round of tests, the earliest instar larvae were seen on day 5, in half of the basins, and the first pupae seen on day 9 in one basin enriched with ^13^C. All the basins produced pupae and collections were done as described below.

### Sample collection and stable isotope analysis

To assess the success of this enrichment-based mosquito marking process, we collected all observed pupae every day from all three sets of wash basins. The collection was done daily and exhaustively i.e. in the morning and evening to ensure no emerged adult escaped. Each collection day, the pupae were put into netting cages allowed to emerge into adults, but a small sub-sample of pupae were kept individually in 2 ml plastic tubes and stored in -20°C freezer for later processing. All emerged adults from each basin were transferred to the laboratory and stored in a freezer at -20°C for further processing.

Once the field sampling collection was complete, all the stored adults mosquitoes were morphologically identified and separated by taxa and sex using taxonomic identification keys [19]. Pools of up to four individual mosquitoes of same sex and species were then prepared and kept in 2ml plastic tubes. The pupae, on the other hand, were arranged singly into the 2ml tubes due to their weight. All the samples were arranged in sample holding boxes, and then dried in an oven at 60°C for 24 hours. To minimize cross contamination, we used different pairs of forceps for handling samples from basins with each of the two enrichment regimens and also different pairs of forceps for the controls. For stable isotope analysis, in round one experiment, mosquito pools consisted of mosquitoes from individual treated basins with the aim to detect the level of enrichment.

In the second round of tests, only adult mosquitoes, but not pupae were assessed. Unlike in the first round where mosquitoes originating from individual basins were analysed separately, the analysis in the second round of experiments involved combining mosquitoes from different enriched or unenriched basins, such that each pool with a maximum of four mosquitoes contained either 0, 1, 2, 3 or 4 individual mosquitoes from enriched basins, the rest being from unenriched basins. This way, we could determine whether the level of enrichment achieved could enable detection of even a single marked mosquito from a pool of four mosquitoes, and therefore assessing the feasibility of scaling-up this stable isotope mark-capture technique to a larger field study.

Samples were analyzed by elemental analysis isotope ratio mass spectrometry (EA-IRMS) to obtain isotopic ratios for carbon (13C/12C) and nitrogen (15N/14N) as previously described [20]. Briefly, each sample was dried, placed into tin capsules, and analyzed on a Carlo Erba NA1500 Series 2 elemental analyzer (EA) coupled to a Finnigan Delta Plus XP (Thermo Fisher Scientific, Waltham, MA) stable IRMS via a Finnigan Conflo III open split interface at the Stable Isotope Geosciences Facility at Texas A&M University. The EA process combusts the samples at 1,020°C, and the resulting CO_2_ and N_2_ gases are separated and analyzed on the IRMS. Results are presented in standard delta (δ) notation δX = [(Rsample/Rstandard) -1] x 1000, where R is the ratio of the heavy to light stable isotope in the sample and standard. Results are referenced to the Vienna Pee Dee Belemnite (VPDB) carbonate standard for δ^13^C, and relative to air for δ^15^N.

### Data analysis

Analysis was performed in SPSS statistical software version 21 [21] and R version 3.2 [22]. We used Wilcoxon-signed rank test to compare mean δX values for mosquito samples emerging from ^15^N-enriched and ^13^C-enriched sites, relative to δX values for samples from controls (non-enriched sites). The adult mosquito samples were grouped by sex and species. For pupae, we used the same test to compare means between treatments and controls, as they were not identified to species level. All the means are presented with associated standard errors, and the significance levels were set at 0.05.

### Ethical approval

This study was part of a broader study on auto-dissemination of insecticides by mosquitoes and was conducted within grounds owned by Ifakara Health Institute. Ethical review and approval was provided by Ifakara Health Institute Review Board (IHI-IRB) (IHRDC/IRB/NO.A-32) and National Institute of Medical Research (NIMR) (NIMR/HQ/R.8a/Vol. IX/764). Desiccated mosquito samples were shipped to the U.S. using United States Department of Agriculture (USDA) import permit number 128949. For inclusion of the participant’s photos for publication of this work, the participants have given written informed consent to publish these details.

## Results

**Fig 2:**
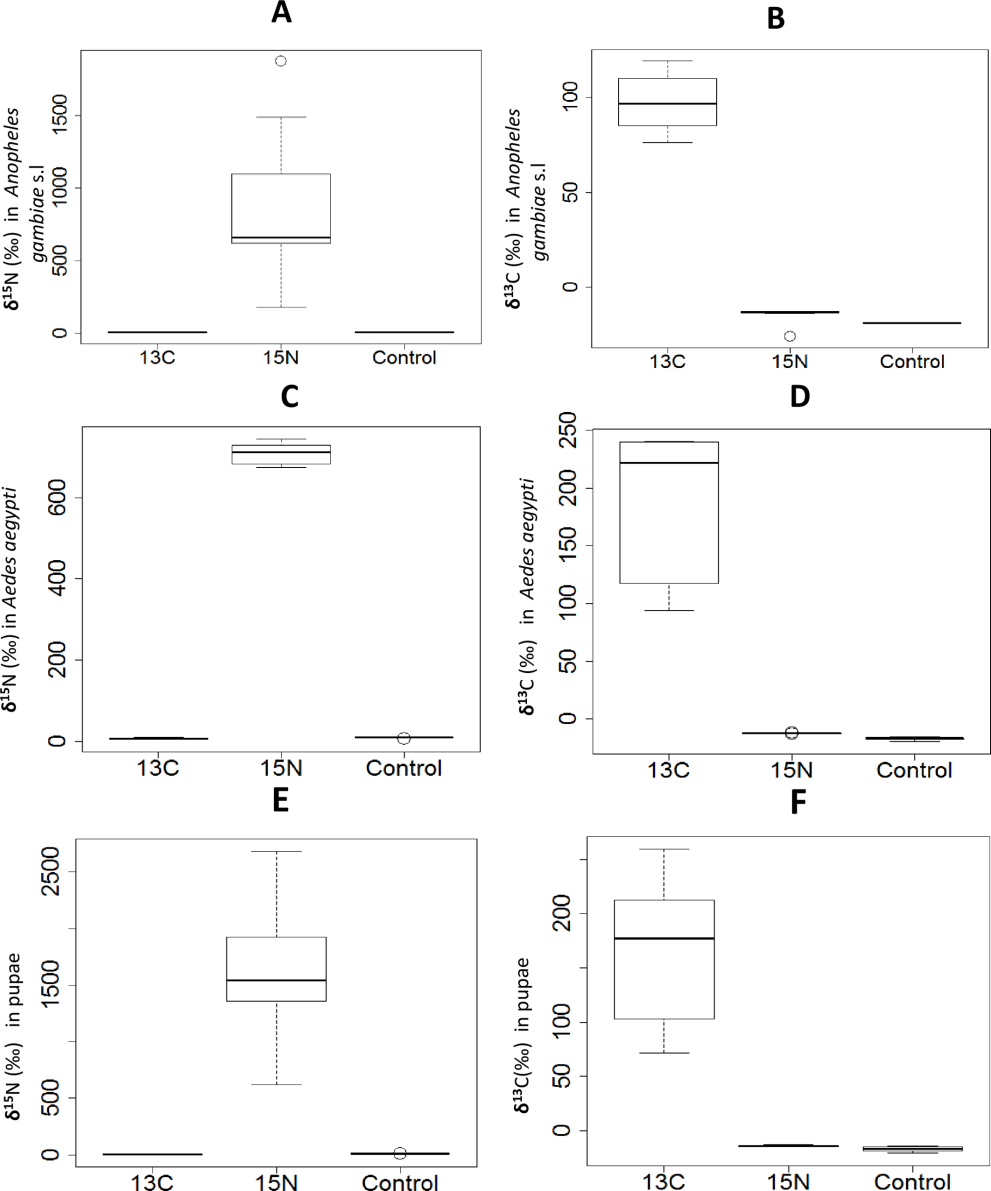
Comparison of isotopic ratios between mosquitoes obtained from enriched pools, and those obtained from control basins: Standardized isotopic ratios δ^15^N and δ^13^C for adult male and female *Anopheles gambiae* sensu lato and *Aedes aegypti*, and the pupae collected from control and enriched basins. Figure panels A, C and E represent results for mosquitoes collected from basins enriched with ^15^N-labelled potassium nitrate, and the respective controls, while figure panels B, D and F represent results of mosquitoes collected from basins enriched with ^13^C-labelled glucose, and the respective controls. All values referenced against international standards (nitrogen = air; carbon = Vienna Pee Dee Belemnite (VPDB).

For round one experiment, the mean δ^15^N and δ^13^C in the controls (representative of natural abundance ratios) were 2.1±0.1 (n = 24 pools) and -4.3±0.2 (n = 24 pools) for females and 3.9±1.7 (n = 13 pools) and -7.9±3.6, (n = 13 pools) for males respectively (Table 1). As shown in Fig 2 & Table 1, emergent adult mosquitoes from pools enriched with ^13^C or ^15^N had significantly higher isotopic levels than adult mosquitoes from non-enriched controls (P = 0.001). Female mosquitoes from habitats enriched with ^15^N–labelled fertilizer had higher isotopic levels than females from habitats enriched with ^13^C-labelled glucose. Both isotopes however produced sufficient distinction between marked and unmarked mosquitoes (Mean δ^15^N for enriched females and males: 275.6±65.1, 248.0±54.6 and Mean δ^15^N in controls; 2.1±0.1, 3.9±1.7 respectively and Mean δ^13^C for enriched females and males; 36.1±5.3, 38.5±6.9 and Mean δ^13^C in controls; -4.3±0.2, -7.9±3.6 respectively).

**Table 1:** Results showing δ^15^N and δ^13^C mean (±SE) of adult male and female pooled mosquitoes regardless of species from habitats enriched using ^15^N-labelled potassium nitrate, ^13^C-labelled carbon and un-enriched habitats (controls).

			δ^15^N		δ^13^C	
	No. of pools	n	Mean±SE	P-value	Mean±SE	P-value
Controls (non-enriched pools)						
Females	6	24	2.1±0.1		-4.3±0.2	
Males	4	13	3.9±1.7		-7.9±3.6	
^15^N-labelled potassium nitrate habitats				0.005		
Females	8	26	275.6±65.1			
Males	8	29	248.0±54.6			
^13^C-labelled glucose habitats						0.003
Females	10	22			36.1±5.3	
Males	6	40			38.5±6.9	

Each pool contained a maximum of 4 mosquitoes; n is the total number of mosquitoes in the pools; δ is the ratio of the isotopes referenced against international reference (nitrogen = air; carbon = Vienna Pee Dee Belemnite (VPDB)) defined by δX = [(R_sample_-R_standard_)/R_standard_] x 1,000 where X is either ^15^N or ^13^C; R_sample_ is the isotopic ratio (^15^N/^14^N, or ^13^C/^12^C) in the sample, R_standard_ is the ratio in the reference; ^15^N and ^13^C are stable isotopes of Nitrogen and Carbon respectively.

**Fig 3:**
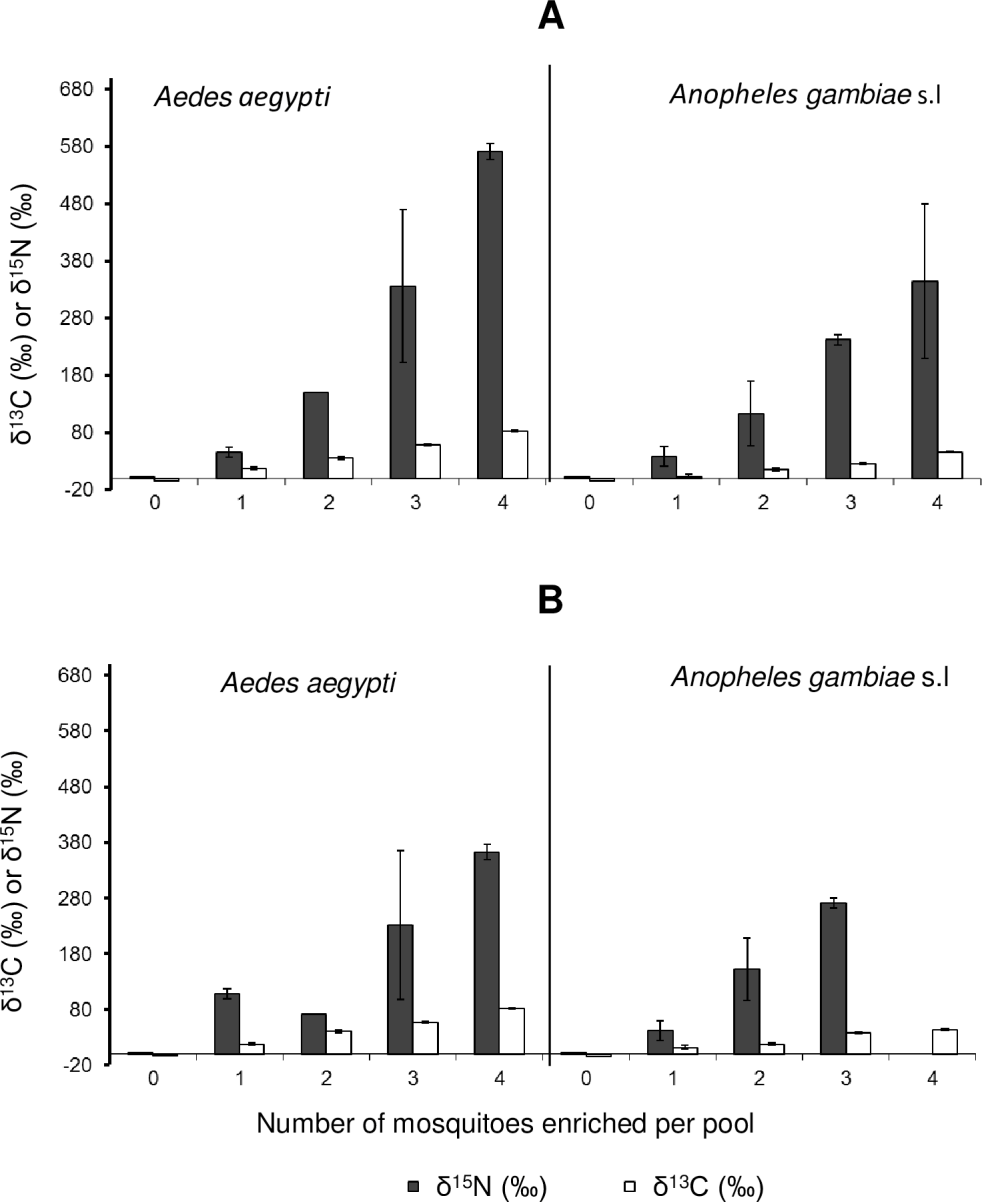
Illustration of how the mean isotopic ratios of δ^15^N and δ^13^C change when different quantities of mosquitoes from enriched versus control basins are included in the pools for analysis. Mean (CI: 95%) δ^15^N and δ^13^C for mosquitoes obtained from basins that were either enriched using the stable isotopes (enriched mosquitoes) or control basins that were not enriched (unenriched mosquitoes). We analysed adult mosquitoes in pools containing a total of four mosquitoes, but variable ratios of enriched to unenriched mosquitoes as indicated by the x-axis label (i.e. 0/4, 1/4, 2/4, ¾ or 4/4). Panel A represents females for *Aedes aegypti* and *Anopheles gambiae* sensu lato, while panel B represents males of the same species. All values referenced against international standards (nitrogen = air; carbon = Vienna Pee Dee Belemnite (VPDB)).

Similarly, pupae collected from habitats enriched with ^15^N–labelled fertilizer and ^13^C-labelled glucose had nearly 4 times higher enrichment levels (mean δ ^15^N of 1624.7±338.7, and mean δ ^13^C of 166.8±28.6) than newly emerged adults from the same basins (Table 2). The mean δ ^15^N and δ ^13^C for pupae from control were 8.8±0.7 and -17.03±1.0, while the mean δ ^15^N and δ ^13^C from treatment were 1624.7±338.7 and 166.8±28.6 respectively (Table 2). The malaria vectors, *An*. *gambiae* s.l. had higher mean levels of ^15^N enrichment than *Aedes* spp. which were highly enriched in ^13^C levels (Table 2). In the second round experiment, we also observed that the mean δ^15^N and δ^13^C was significantly higher in any pool containing at least one mosquito from enriched basins, compared with the control pools, i.e. pools in which all mosquitoes were from non-enriched basins (P<0.001), Fig 3. The mean δ^15^N and δ^13^C increased with increasing number of mosquitoes from enriched basins, included in each pool (Fig 3).

**Table 2:** Mean (±SE) isotopic ratio (δ) of mosquitoes obtained from habitats enriched using ^15^N-labelled potassium nitrate and ^13^C-labelled glucose. All values referenced against international standards (nitrogen = air; carbon = Vienna Pee Dee Belemnite (VPDB)). Data grouped by species and sex for adult mosquitoes and combined pupae.

				δ^15^N			δ^13^C		
Treatment	Mosquito	Sex	No. of pools	n	Mean±SE	P-value	n	Mean±SE	P-value
Untreated habitats (Control)	*Aedes spp*.	F	6	24	2.4±0.2			-4.3±0.2	
	*Aedes* spp.	M	4	16	2.6±0.3			-4.2±0.0	
	*An*. *gambiae* s.l	F	2	8	3.0±0.0			-4.5±0.1	
	*An*. *gambiae* s.l	M	3	9	5.1±2.0			-9.2±4.7	
	*An*. *maculipalpis*	F	2	8	2.1±0.3			-4.2±0.0	
	*An*. *maculipalpis*	M	1	4	2.0±0.0			-4.4±0.0	
	Pupae		7	7	9.0±0.7			-7.03±1.0	
^13^C-enriched habitats	*Aedes spp*.	F	12				48	49.7±6.5	0.005
	*Aedes spp*.	M	8				32	45.1±21.4	
	*An*. *gambiae* s.l	F	14				56	23.9±3.4	
	*An*. *gambiae* s.l	M	6				22	29.3±6.2	
	Pupae						6	166.8±28.6	
^15^N-enriched habitats	*Aedes spp*.	F	12	48	244.7±51.7	0.005			
	*Aedes spp*.	M	8	32	182.5±30.9				
	*An*.*gambiae* s.l	F	12	42	246.0±53.7				
	*An*.*gambiae* s.l	M	8	27	264.0±63.4				
	Pupae		5	5	1624.7±338.7				

## Discussion

This study was primarily designed to demonstrate use of stable isotope enrichment technique for marking *Anopheles* and *Aedes* mosquitoes at their larval habitats. The findings suggest sufficient marking of wild malaria mosquitoes, *An*. *gambiae* s.l. but also the dengue vectors *Ae*. *aegypti*, which constitute nearly all the *Aedes* species found in the area, through larval source enrichment. Stable isotopes were incorporated and fixed into the larval tissues and was retained to detectable levels in newly emerged adults. By adding 99 atom% ^15^N and 99 atom% ^13^C, sufficient enrichment was achieved, that was significantly above the natural abundance of field mosquitoes (controls) [11]. We found higher isotopic levels in mosquitoes with nitrogen than carbon (Figs 2 and 3). This is likely because nitrogen is fixed slowly into the structural tissues such as chitin and has a very low rate of turnover than carbon [10,11]. Our study also demonstrates that it is possible to detect whether mosquitoes originating from basins or areas with enrichment, even if only very few of these mosquitoes are captured per sample, corroborating previous studies [4]. This information is particularly important to inform field studies where dilution effect is likely to occur.

The results also show that both *Anopheles* and *Aedes* spp. achieved higher levels of marking with stable isotopes of nitrogen than carbon, suggesting perhaps higher dependence on nitrogen rich food resources [15], (Figs 2 and 3). This may also suggest ^15^N would be preferably useful to mark malaria vectors, even though ^13^C-labelled glucose also yielded clear distinctive differences between treatment and controls. Our results also demonstrate that pupae had higher mean enrichment levels of nitrogen and carbon than newly emerged adult mosquitoes (Table 2). However, this is in contrast with previous findings by Hamer *et al*. 2012 [4] who showed no difference in level of enrichment between pupae, 4^th^ larvae instar and newly emerged adults. This difference might be attributed to eclosion difference among mosquito species. We did not investigate the effects of stable isotope enrichment on mosquito behaviour or survivorship, although previous studies have not found major differences in *Culex* mosquitoes achieving similar levels of enrichment [4]. The effect of stable isotope enrichment on *Anopheles* spp. mosquitoes warrants further attention. Mosquito marking observed in this study might have occurred due to the enriched nitrogen and carbon fixed (bioaccumulation) into larval diet such as algae, bacteria and other microorganism which they feed on [23].

This work validates earlier observations that habitat enrichment with stable isotopes offers an effective alternative marking technique for medically important mosquitoes [10]. The technique can also be used to complement already existing mark-recapture techniques. The technique is advantageous in that large numbers of mosquitoes can be marked at once and with minute amounts of enriched compounds. For example in our study, we used only 0.3g of ^13^C-labelled glucose and 0.3 g of ^15^N-labelled potassium nitrate to enrich mosquito larval habitats that produced nearly 3000 adult mosquitoes. This technique can as well be implemented to enrich naturally breeding mosquitoes from their larval habitats. For example, previous study by Hamer *et al*. 2014 [13] implemented field stable isotope enrichment technique to investigate dispersal range of *Culex* spp. mosquitoes, the vectors of West Nile Virus (WNV). Mosquito species which breed in small and confined larval habitats such as *Aedes* spp. and other *Culex* spp. can easily be enriched with stable isotopes. For *Anopheles spp*. such as *An*. *gambiae* which breed in large bodies of water, enrichment could be deployed by targeting areas most preferred by larvae through spraying of the isotopes or during dry season, when aquatic habitats are geographically restricted and reduced in size.

The approach that we have validated in this study could significantly enhance studies on mosquito eco-physiology, dispersal and the role of these vectors in pathogen transmission. Moreover, field evaluation of vector control strategies, including novel techniques such as auto-dissemination of insecticides by adult mosquitoes into their larval habitats [24] could utilize stable isotope enrichment marking technique to assess the intervention effectiveness. Such evaluation could be done by marking the intervention and control larval habitats with different markers (e.g. stable isotopes of carbon and nitrogen) then assessing the movement of mosquitoes beyond the targeted sites and how such movements attenuate efficacy of the candidate vector control strategy. By using different stable isotopes to mark mosquitoes in different villages undergoing specified interventions, we could determine whether there is any encroachment between villages and whether the interventions are achieving the necessary effects locally. One disadvantage of the stable isotope marking technique is the cost of analysis for each sample. However, for large field implementation this cost is minimized by the ability to test mosquitoes in pools containing multiple individuals. Another disadvantage of this technique is that, it is not easy to determine the age structure of wild mosquito population since release of mosquitoes occur in undefined time making it difficult for follow up in the field. With regard to the specific stable isotopes used in this study, it should also be noted that since the breakdown of ^13^C is higher due to respiration and other biochemical processes, further evaluation of the quantity of ^13^C required to enrich natural larval habitats without complete loss into the system is needed. Studies are therefore also needed to evaluate how long the larval habitats remain enriched especially in the field settings and the retention of stables isotopes post emergence in malaria and dengue vectors in the field using a similar set up in the current study.

## Conclusions

Our study demonstrates that enrichment of larval habitats using stable isotopes of carbon (^13^C) and nitrogen (^15^N) was sufficient to distinguish between *Anopheles* and *Aedes* mosquitoes originating from enriched habitats and those originating from control non-enriched habitats, which had only natural abundance levels of these isotopes. Labelling mosquitoes in the field was easy, simple and straight-forward. Naturally occurring stable isotopes of nitrogen and carbon can therefore be efficiently and easily used for making wild disease-transmitting mosquitoes. We conclude that this approach can significantly enhance studies on mosquito eco-physiology, dispersal, pathogen transmission and responses to control measures, including interventions against the Afro-tropical malaria vectors.
